# Crowdsourced Health Research Studies: An Important Emerging Complement to Clinical Trials in the Public Health Research Ecosystem

**DOI:** 10.2196/jmir.1988

**Published:** 2012-03-07

**Authors:** Melanie Swan

**Affiliations:** ^1^MS Futures GroupPalo Alto, CAUnited States

**Keywords:** Community-Based Participatory Research, Preventive Medicine, Personalized Medicine, Individualized Medicine, Consumer Participation, Health Services Research, Health Care Research, Public Health, Genomics, Medicine

## Abstract

**Background:**

Crowdsourced health research studies are the nexus of three contemporary trends: 1) citizen science (non-professionally trained individuals conducting science-related activities); 2) crowdsourcing (use of web-based technologies to recruit project participants); and 3) medicine 2.0 / health 2.0 (active participation of individuals in their health care particularly using web 2.0 technologies). Crowdsourced health research studies have arisen as a natural extension of the activities of health social networks (online health interest communities), and can be researcher-organized or participant-organized. In the last few years, professional researchers have been crowdsourcing cohorts from health social networks for the conduct of traditional studies. Participants have also begun to organize their own research studies through health social networks and health collaboration communities created especially for the purpose of self-experimentation and the investigation of health-related concerns.

**Objective:**

The objective of this analysis is to undertake a comprehensive narrative review of crowdsourced health research studies. This review will assess the status, impact, and prospects of crowdsourced health research studies.

**Methods:**

Crowdsourced health research studies were identified through a search of literature published from 2000 to 2011 and informal interviews conducted 2008-2011. Keyword terms related to crowdsourcing were sought in Medline/PubMed. Papers that presented results from human health studies that included crowdsourced populations were selected for inclusion. Crowdsourced health research studies not published in the scientific literature were identified by attending industry conferences and events, interviewing attendees, and reviewing related websites.

**Results:**

Participatory health is a growing area with individuals using health social networks, crowdsourced studies, smartphone health applications, and personal health records to achieve positive outcomes for a variety of health conditions. PatientsLikeMe and 23andMe are the leading operators of researcher-organized, crowdsourced health research studies. These operators have published findings in the areas of disease research, drug response, user experience in crowdsourced studies, and genetic association. Quantified Self, Genomera, and DIYgenomics are communities of participant-organized health research studies where individuals conduct self-experimentation and group studies. Crowdsourced health research studies have a diversity of intended outcomes and levels of scientific rigor.

**Conclusions:**

Participatory health initiatives are becoming part of the public health ecosystem and their rapid growth is facilitated by Internet and social networking influences. Large-scale parameter-stratified cohorts have potential to facilitate a next-generation understanding of disease and drug response. Not only is the large size of crowdsourced cohorts an asset to medical discovery, too is the near-immediate speed at which medical findings might be tested and applied. Participatory health initiatives are expanding the scope of medicine from a traditional focus on disease cure to a personalized preventive approach. Crowdsourced health research studies are a promising complement and extension to traditional clinical trials as a model for the conduct of health research.

## Introduction

Crowdsourced health research studies are the nexus of three contemporary trends: citizen science (non-professionally trained individuals conducting science-related activities), crowdsourcing (use of web-based technologies to recruit project participants), and medicine 2.0 / health 2.0 (active participation of individuals in their health care particularly using web 2.0 technologies). Crowdsourced health research studies have arisen as a natural extension of the activities of online health social networks and communities. Studies may be researcher-organized or participant-organized. Professional researchers crowdsource cohorts from health social networks for the conduct of traditional studies. In contrast, participants organize their own health research studies through health social networks and health collaboration communities; these communities are created for the purpose of self-experimentation and for the investigation of shared health concerns together in groups. Before embarking on a narrative overview of crowdsourced research studies, I will first consider the definition of citizen science, crowdsourcing, medicine 2.0 / health 2.0, and crowdsourced health research studies. I will then provide an overview of crowdsourced health research studies that have been conducted by professional researchers and/or participants. Finally, I will discuss the limitations of these methods, and offer conclusions.

### Citizen Science

Citizen science is the conduct of science-related activities by individuals who have no formal training in a field specific to the topic of investigation. Citizen science practitioners may include laypersons, scientists, or professionals trained in other fields. Citizen science projects have been in existence for hundreds of years; the professional scientist is a relatively recent incarnation. One prominent example of citizen science is the National Audubon Society’s annual Christmas bird watch, in its 112th year with tens of thousands of participants in 2011 [1]. Another high-profile project is Galaxy Zoo, where over 250,000 individuals have annotated astronomical data from the Sloan Digital Sky Survey, surprising project organizers by completing 50 million images in the first year as opposed to an anticipated 1 million [2]. A citizen science referral and advocacy website, SciStarter [3], listed 340 projects for participation as of January 2012 in 20 areas ranging from the environment to health. The Citizen Science Alliance [4] is another industry group which supports citizen science and coordinates Galaxy Zoo and other astronomy-related projects.

### Crowdsourcing

Crowdsoucing is the practice of obtaining participants, services, ideas, or content by soliciting contributions from a large group of people, especially via the Internet [5]. Canvasing vast numbers of individuals through an open call facilitates self-selection. In particular, there is potential for crowdsourcing to capitalize on the input of interested and fit individuals who have the best ideas and bring a diverse set of skills and backgrounds to bear on the current task. Some notable examples of successfully crowdsourced projects are discussed in *The Wisdom of Crowds* [6] and *Wikinomics* [7].

### Medicine 2.0 / Health 2.0 and Participatory Medicine

Some of the early definitional discussions of medicine 2.0 / health 2.0 (which are largely used synonymously [8]) occurred in 2008 and focused on the deployment of social media in the health context; that is, that medicine 2.0 / health 2.0 is the use of web 2.0 tools (eg, blogs, podcasts, tagging, search, wikis, video) by health care actors to improve collaboration and personalize health care [8,9]*.* In 2010, a related concept, participatory medicine, was introduced to emphasize the active participation of individuals: “This new definition devised by the board of the Society of Participatory Medicine is a movement in which networked patients shift from being mere passengers to responsible drivers of their health, and in which providers encourage and value them as full partners [10]*.*”

These definitions have helped to undergird the medicine 2.0 / health 2.0 and participatory medicine movement. A Pew Internet study found that 27% of US Internet users had tracked health data online and 18% had sought to locate others with similar health concerns via the Internet [11]. At present, individuals have the opportunity to self-manage their health using web 2.0 tools, smartphone health applications, online personal health records, and health social networks. Health social networks, essentially Facebook or LinkedIn for health interest areas, are online communities where individuals may find and discuss information about conditions, symptoms, and treatments; provide and receive support; enter and monitor data; and join health studies [12]. Health social networks exemplify the predicted progression of engagement in online communities, escalating in three stages from information-sharing, to cooperating, to participating in collaborative action [13]. As of January 2012, some of the largest health social networks for patients are MedHelp (claiming over 12 million monthly visitors), PatientsLikeMe, DailyStrength, Tudiabetes, CureTogether, and Asthmapolis; and for physicians, Sermo, Ozmosis, and RadRounds [14].

### Crowdsourced Health Research Studies 

The nexus of these trends—citizen science, crowdsourcing, and medicine 2.0 / health 2.0—is crowdsourced health research studies. One indication of the relative newness, growth, and interest in this area is the exponential rise in recent Internet activity in crowdsourced health research. In 2011, 1,920,000 results were returned for a Google search of the terms ‘crowdsourcing and health’; in 2010 and 2009 the comparative figure was 669,000 and 318,000 respectively. In January 2012, the term ‘crowdsourcing’ in a PubMed search yielded 16 publications, 13 of which were published in 2011.

Crowdsourced health research studies may be a blend of crowdsourcing and citizen science. In addition, these terms are often used interchangeably. This lack of precision in language is an artifact of the relative newness of these concepts that are being defined through use. Within crowdsourced studies, participants are recruited via crowdsourcing (eg, recruited online with a website or an open call to a large potential audience using Internet-related technologies). In crowdsourced studies participants could be subjects not performing any science-related activities themselves, and therefore would not be considered citizen scientists. The use of the term ‘citizen scientist’ denotes the conduct of science-related activities by participants. Examples are citizen scientists being crowdsourced to annotate astronomical data in the Galaxy Zoo projects, or when an online crowd analyzed scientific images of cells in a tuberculosis study [15]. There may be many other permutations. Citizen scientists might be crowdsourced for idea generation and hypothesis formation, data collection, results analysis, results dissemination, and/or study funding (‘crowdfunding’).

In addition to opportunity for participant engagement, crowdsourced research may be different to traditional studies in other ways. First, crowdsourced research provides opportunity for more levels of openness and privacy, as participants decide what data to share with whom. One potential result is that there is less regulated protection of research subjects. Instead individuals take responsibility for informing themselves (possibly in consultation with physicians) about self-experimentation or study participation. Second, within crowdsourced research the rewards may accrue more directly to study participants and health communities as opposed to study funders in the more traditional model. Third, funding may come from alternative sources such as academia, industry, patient advocacy groups, research foundations, social venture capital, crowdfunding, and self-funding.

In this article, the term ‘crowdsourced health research studies’ is used to indicate that health study participants are recruited with crowdsourcing (eg, Internet-based) techniques. Study participants may or may not be acting as citizen scientists (ie, conducting science-related activities).

#### Participant Motivations and Expectations

Individuals have a variety of motivations for participating in crowdsourced health research studies. On a personal level, they may be drawn by natural curiosity, wanting to tinker and test hypotheses in a health interest area. Individuals taking a broader societal perspective may wish to participate in, contribute to, impact on, and at times conduct, projects that are outside the scope of traditional research. Another dimension of the broader societal perspective is how individuals may view themselves in relation to society. There is a developing notion of biocitizenry: that being a citizen scientist, and sharing personal health information, or using it as a currency for gaining access to studies could be considered acts of citizenship [16,17].

The direct and personal connection that individuals have to health makes crowdsourced health research distinct from other crowdsourced studies. Perhaps due to this personal connection, and the ease of self-tracking and experimenting with interventions, the role of the participant is expanding more quickly in health compared to other citizen science areas. In particular, the participant is engaging not just as a provider of outsourced data collection, but also helping with data analysis, and possibly the design and conduct of studies. Participant expectations of their engagement in crowdsourced studies are also different as they make demands on study organizers to return study data, provide interpretive personalized recommendations, and want the ability to communicate with other study participants [18].

#### Types and Methods of Crowdsourced Health Research Studies

Crowdsourced health research studies can be researcher-organized or participant-organized. Researcher-organized studies are typically traditional studies organized by institutionally-trained researchers using crowdsourced health social network cohorts or crowdsourced data as the input or research focus; for example, studies organized by PatientsLikeMe [19] and 23andMe [20]. Participant-organized studies are usually designed and operated by citizen scientists; for example, those conducted by PatientsLikeMe patients, DIYgenomics citizen scientists [21], and Quantified Self individual experimenters [22]. 

The research methods available in crowdsourced health research studies are detailed in Table 1.

**Table 1:** Research methods employed to date in crowdsourced health research studies.

	Study organizer	Research methods and types of data available
**Researcher-organized studies**		
	PatientsLikeMe	Self-reported data, survey questionnaires
	23andMe	Genotyping data, survey questionnaires
**Participant-organized studies**		
	Genomera, Althea Health, DIYgenomics	Genotyping data, blood test result PDF files, self-reported data, survey questionnaires
	Quantified Self	Self-tracking device data (eg, myZeo, FitBit, TelCare, etc.), self-reported data (manually collected)

### Principal Aim of This Study

The principal aim of this analysis is to provide an overview of crowdsourced health research studies. The narrative will characterize the nature of current activity and highlight differences between crowdsourcing and traditional methodologies.

## Methods

An analysis of crowdsourced health research studies was undertaken through a literature search and interviews. The literature review consisted of first generating a list of potential published studies for inclusion by searching the keyword terms ‘crowdsourcing, crowdsourced, patient-organized, participatory, self-experimentation, PatientsLikeMe, and 23andMe’ in Medline/PubMed, ISI Web of Science, and Google Scholar. Further searches in the same engines were then conducted for other papers by authors of the publications found in the initial search. Additional papers were also selected from the bibliographies of initially retrieved articles. Searches were conducted of papers published from 2000 to 2011. The inclusion criteria were that papers needed to report on (1) a human health study, (2) a study conducted on a crowdsourced population, and (3) a study with protocol details and results. Crowdsourced health research studies not published in the scientific literature were identified by attending 5 larger conferences (Medicine 2.0 congress, also known as the World Congress on Social Media, Mobile Apps, and Web 2.0 in Health and Medicine [23], Quantified Self, HealthCamp [24]) and over twenty Quantified Self meetups [22] in different cities from 2008 to 2011. Discussions and follow-up discussions were held with event participants, and related websites were reviewed (Medicine 2.0 [23], Quantified Self [22], Genomera [25], Althea Health [26], and DIYgenomics).

## Results

### Researcher-Organized Studies: PatientsLikeMe, 23andMe

#### PatientsLikeMe Crowdsourced Studies

**Figure 1:**
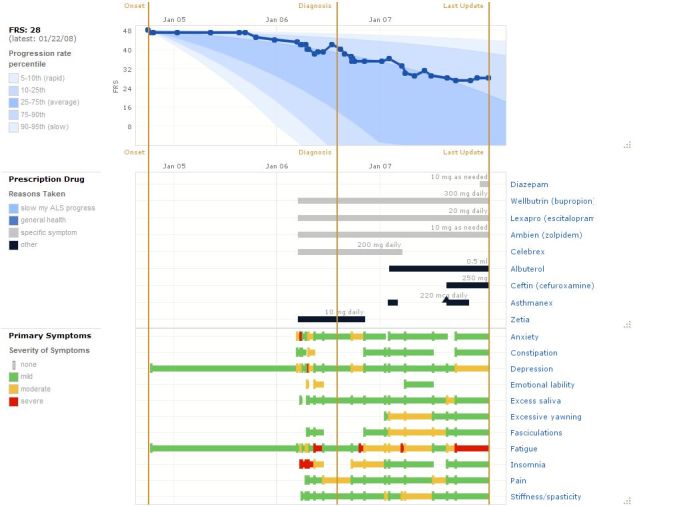
Charts comprising the personal profile of a user on PatientsLikeMe (Image Source: Frost & Massagli, Journal of Medical Internet Research [74], licensed under Creative Commons Attribution License 2.0).

PatientsLikeMe (PLM) is currently the largest operator of crowdsourced health research studies with one of the largest open patient registries and online health social networks (more than 125,000 members in 1000 condition-based communities as of January 2012). Amongst other initiatives, the company aims to connect 1 million rare disease patients by the end of 2012 [27]. Members may enter demographic information and track their treatments, symptoms, and outcomes, and find other patients like themselves matched by demographic and clinical characteristics (see Figure 1). Over 25 PLM-authored papers have been published in peer-reviewed journals such as the *Journal of Medical Internet Research*, *Nature Biotechnology*, the *Proceedings of the National Academy of Sciences*, and recognized neurology journals, many of which present the results of researcher-organized crowdsourced studies.

One of the best known PLM studies is the lithium study [28], which is also an interesting model of how patient-organized crowdsourced studies, researcher-organized crowdsourced studies, and traditional randomized controlled trials (RCTs) may be complementary phases in the overall investigatory process. In one of the first reported cases of patient-organized studies, a PLM community member with amyotrophic lateral sclerosis (ALS) found a small blinded Italian study (16 cases and 28 controls) [29] where lithium was found to slow disease progression in ALS patients, but which also warned that the model might not be applicable in other circumstances. The PLM community member convinced others to collaborate in a participative study where patients would apply the published findings in the Italian study to themselves. Initially, 348 PLM patients began the off-label use of lithium, overseen by their physicians. At the end of the study, self-reported data were available for 149 patients who took lithium for at least two months, and 78 patients who took lithium for 12 months. Ultimately, lithium was found not to have a positive impact in slowing disease progression in ALS patients in three tiers of study: initially through PLM patient self-experimentation, then through an observational study conducted by PLM researchers by comparing the 149 cases with 447 controls based on disease progression, and later in traditional randomized studies [30,31]. The distinguishing feature of the patient-organized portion of the study that characterizes it as citizen science was the instigative role of the patients in identifying and applying the study to themselves, self-collecting and reporting data, and seeking the drug directly rather than having it sponsored by a drug company. In the future, self-experimentation in citizen science cohorts might act as a real-time sensor network or barometer for early indications that could be later confirmed in more structured studies or RCTs.

##### Amyotrophic Lateral Sclerosis (ALS)-Specific Research

ALS is PLM’s flagship community and a key research area. A recent study used questionnaires to investigate a potential connection between the physical use of a limb and disease onset, and found that there was concordance for handedness but not footedness in the limb onset of ALS in 343 patients. The study found that this could be due to 1 arm typically dominating in upper-body activities but both limbs being used equally in lower-body activities such as standing and walking. Cortical factors could also be related [32]. Another study used questionnaires, taking the standardized disease measurement scale for ALS as a starting point for improving the lack of detailed measures of patient function sensitivity in advanced ALS. Of 10 new items investigated, 3 were suggested for inclusion in the scale: the ability to use fingers to manipulate devices, show emotional expression in the face, and get around inside the home [33]. Other work employed questionnaires to determine some of the reasons for low participation in ALS studies, and found patients are not being invited to enroll, have concerns about the cost of participation, and are confused over aspects of the studies [34].

One benefit of the new crowdsourced methodologies, having over 1000 conditions on the PLM platform for example, is the ability to conduct comparative research. One study identified that the tendency towards pathological gambling in Parkinson’s disease patients also may exist in ALS patients, although to a lesser degree (13% versus 3% respectively) [35].

##### Health Social Network Findings Related to Prescription Drug Use

Health social networks can be a useful resource for investigating drug-related activity such as off-label use, side effects, product safety, and patient sentiment. Off-label drug use is commonplace (21% of US prescriptions) as physicians may use a side effect as a main effect. However, scientific evidence for off-label drug use is lacking for 73% of cases, and wasteful or harmful treatment may occur [36]. Additionally, prescribers may not have enough cases or appropriate experimental processes to establish statistically meaningful off-label use. The larger numbers of patients available in health social networks can help in a more systematic investigation of off-label drug use. In one study, PLM analyzed off-label drug use for amitriptyline and modafinil, respectively approved for treating depression, and narcolepsy or sleep apnea. The study found that 91% (n = 1089) of amitriptyline users took the drug for an off-label use, as did 99% (n = 1737) of modafinil users. The off-label use was specific to disease conditions and showed a benefit. Taking advantage of a normally unpleasant dry mouth side effect, 40% of amitriptyline users with ALS reported a reduction in unwanted excess saliva. Likewise, 36% of wakefulness-promoting agent modafinil users with multiple sclerosis (MS) and Parkinson’s disease reported an improvement in combating the general fatigue of the conditions. It was concluded that patient-reported outcomes could provide a new source of evidence about secondary uses for drugs and potentially identify targets for further study in RCTs.

In another PLM study, information reported online by the MS community was used to develop a survey for quantifying medication adherence, a known challenge particularly with MS [37]. Of the 36% of the PLM MS community that participated in the survey, 16% to 51% (depending on the treatment) of patients reported missing at least one dose of medication in the last 28 days. User-reported information like this could be used to develop more effective medication regimens based on user behavior by being more reflective of disease cycles and the daily rhythms of patients. Regarding perceptions of product safety, another study found that patient sentiment (per PLM forum discussion) remained positive for the MS drug Tysabri (natalizumab) even after it was linked to 3 cases of progressive multifocal leukoencephalopathy in 2008 [38].

##### Experience of Individuals Participating in Health Social Networks

In addition to the conduct of condition-related research, PLM examines the ongoing user experience of health social network participation. In one study, 19% of PLM community members responded to a survey, and when queried, overwhelmingly reported having had a positive experience with health social networking. Members used the site to learn about symptoms, understand treatments and their side effects, and make decisions about treatments (for example to start or stop treatments or change a dosage). Members also reported increased comfort in sharing personal health information [39]. 

Another study reports on the benefits of obtaining information about disease peers in health social networks. Benefits include the ability for patients to know how well they are doing in comparison to others and if they are receiving the most successful treatments [40]. Other studies discussed some of the next steps for improving the quality of information derived from health social networks, for example, having appropriate means of interpreting unstructured information, managing churning community populations, and confirming the accuracy of self-reported data. An effort should be made to determine whether health social network participation improves real-world outcomes, and to identify new tools that could further empower patients in managing their health [38]. One such tool would be expanding the functionality of health social network platforms to facilitate patient-organized studies like the lithium study [36]. 

#### 23andMe Genome Association Studies

23andMe is the largest personal genotyping community, and as of June 2011 had over 100,000 genome service subscribers. A handful of research studies have been published in peer-reviewed journals such as PLoS Genetics and PLoS One. Over 75% of the 23andMe community has indicated a willingness to participate in research studies organized by the company [41,42]. One study was the largest case-control genome-wide association examination of Parkinson’s disease conducted on a single collection of individuals (3426 cases and 29,624 controls). The study replicated 20 previously discovered genetic associations and discovered 2 new ones (rs6812193 near lysosome protein-related SCARB2, and rs11868035 near sterol regulation-related SREBF1/RAI1) [43].

Another study addressed the problem of collecting phenotypic data for large cohorts. 23andMe community members were asked to complete questionnaires, and 20,000 individuals reported data on 50 medical phenotypes. One hundred and eighty previously reported associations (curated by the National Human Genome Research Institute) were replicated for conditions such as type 2 diabetes, prostate cancer, cholesterol levels, and multiple sclerosis. These were, however, only 75% of expected associations [44], underlining the challenges of applied genetics [45] and suggesting the potential value of large-cohort follow-on studies.

An earlier study validated self-reported data in health social networks, focusing on non-disease conditions. Existing genetic associations were replicated for hair color, eye color, and freckling, and novel associations were found for hair morphology, freckling, smell detection, and sneeze reflex [46]. In addition to targeted studies, 23andMe has a community research effort, 23andWe, with ongoing open-enrollment via their website for a variety of conditions such as Parkinson's disease, sarcoma, and myeloproliferative neoplasms [47].

### Participant-Organized Studies: Quantified Self, Genomera, and DIYgenomics

Self-experimentation and participant-organized studies may arise naturally through health social networks like the PLM lithium study, and also through communities that have been created expressly for the purpose of conducting experiments in individuals and groups. One such example is the Quantified Self community, a collaboration of users and toolmakers who share an interest in personalized knowledge through self-tracking [48]. The initial meeting of the group was on September 10, 2008 in San Francisco, California with 28 attendees. Just 3 years later, as of January 2012, 5524 members were listed in 42 worldwide meetup groups [22]. Numerous self-tracking projects have been shared at ‘Show and Tell’ meetings and approximately 20 posters were presented at the group’s inaugural conference held in Silicon Valley in May 2011 [49]. Another self-tracking community is being convened at an Association for the Advancement of Artificial Intelligence meeting entitled Self-tracking and Collective Intelligence for Personal Wellness, in March 2012 [50], with the conceptual framing of citizen scientists comprising a new form of intelligence, ‘community computing.’

Quantified self-experimentation has produced at least two peer-reviewed journal articles that provide examples of a robust and limitation-cognizant framework for self-experimentation in the areas of sleep, mood, health, and weight [51,52]. Specifically, an individual was able to reduce early awakening by avoiding breakfast and spending more time during the day standing, to improve mood by seeing faces in the morning, and to lose weight by drinking sugar water [51].

For a design school thesis project that was published online but not peer-reviewed, another quantified self-tracker measured several aspects of daily life and presented data visualizations of a year of food consumption [53]. Most quantified self-projects to date have focused on experimentation in n = 1 studies, but group studies are also emerging. One example is the Butter Mind study, which was conducted with the advice of a scientist but not independently reviewed. The results were presented in a blog entry [54]. The randomized experiment had 45 individuals, took place from October 23 to November 12, 2010 and found that eating 2 ounces (56.7 grams) of butter per day resulted in improved arithmetic speed. There were limitations, including no clear articulation of method, a small sample size with limited statistical power, and results that did not control for IQ or education levels. Another crowdsourced group study, the Blueberry Study, has been running since 1999 with hundreds of participants investigating a potential link between blueberry consumption and enhanced mental performance. In 2011, study organizers reported that a 1% improvement in memory performance (as measured by online word recall exercises) occurred within a 1-year period. Results appear to be unreviewed and are reported in conference posters and online [55].

Group studies are also being conducted by DIYgenomics. The organization is attempting to realize preventive medicine by organizing studies according to the generalized hypothesis that one or more genetic polymorphisms may lead to out-of-bounds phenotypic biomarker levels, and that these may be ameliorated through personalized intervention. The methodology and results of a pilot study were published in the *Journal of Participatory Medicine* [56]. In the pilot study, conducted from June to December 2010, a crowdsourced cohort examined the potential role of methylenetetrahydrofolate reductase (MTHFR) polymorphisms in vitamin B deficiency and homocysteine levels, and tested a series of supplement interventions. The study found that 57% (n = 4) of the healthy cohort members already had higher-than-recommended homocysteine levels at baseline, and that personalized vitamin supplementation strategies quickly helped to bring these levels back into recommended ranges, particularly for those that were vegan or vegetarian and had one or more genetic polymorphisms. The best intervention for 5 individuals was the regular B vitamin, and for the 2 remaining individuals was the active form of B-9 (folate). The study was unique in that participants were also investigators, acting in collaboration with each other to determine all aspects of the study including protocol design and results interpretation. In this case, participants wished to be, and were identified publicly by name. Advice on the study protocol was gained from 2 independent experts in the field. The study is ongoing, with open enrollment; there are 24 participants as of January 2012 [57].

DIYgenomics currently has 6 other studies in open enrollment, covering a range of conditions including vitamin deficiency, aging, mental performance, and epistemology. One study concerns memory filtering and is in collaboration with the University of Geneva [58].

#### Study Operation Platforms

The Butter Mind study and the DIYgenomics studies were run on the Genomera personal health collaboration platform. Genomera is conceptually an ‘eBay for health science experiments,’ where any community member (professional researchers and citizen scientists alike) may post a study in an area of interest and attempt to crowdsource participants. As of January 2012, there were 20 studies listed on the site, with enrolled participants ranging from 10 to 60 per study, and over 300 community members volunteering to participate in crowdsourced studies by providing genotypic and phenotypic information. Individuals may participate at different levels with accompanying security protections, for example as a community member, study discussion participant, study data participant, or study organizer. Althea Health is a similar platform for the operation of crowdsourced longitudinal health research studies [26].

#### Self-Tracking Tools

In addition to Internet-based platforms for the automated operation of crowdsourced health research studies, self-tracking tools and their validation and calibration are essential for accurate data collection. The Quantified Self website lists over 400 such tools [59]. Zeo, the provider of a low-cost sleep tracking wireless system, commissioned a study with 29 subjects that validated data collection with their device as compared to traditional sleep laboratory measurements with polysomnography and actigraphy [60]. As widespread low-cost access to automated data collection tools grows, the number of individuals self-tracking and monitoring their health behavior could increase substantially. Continuously collected data from wireless sensors, accelerometers, gyroscopes, and pressure-sensitive textiles could be transmitted via smartphone or home WiFi networks and interpreted into personalized recommendations via the Internet with machine-learning algorithms [61]. Self-tracking data may be useful both to individuals for health self-management, and in clinical trials to convey a richer and less artificial picture of real-life activities.

## Discussion

### Principal Results

Different kinds of crowdsourced health research studies have been conducted. PatientsLikeMe and 23andMe are the leading operators of crowdsourced health research studies and have published results in peer-reviewed journals. Research findings have been in the areas of improving the characterization and measurement of disease, investigating aspects of prescription drug use (off-label use, medication adherence, and patient sentiment), exploring health social network user experience, and establishing disease and trait-based genetic associations. Quantified Self, Genomera, and DIYgenomics are the largest communities of participant-organized health research studies.

Results have been published in peer-reviewed journals, but more often in unreviewed gray literature. Self-experimentation studies have focused on optimizing physical and mental performance, improving alertness, mood, and weight loss, visualizing food consumption, and validating a consumer sleep-tracking device. Collaborative group studies have targeted mental performance, vitamin deficiency, and aging.

### Limitations of Crowdsourced Health Research Studies and Their Critiques

Two dimensions of limitations are considered here. First, limitations in the evidence base (study methods, study design, regulation, and oversight), and second, limitations in critiques made of the field (degree of novel findings, citizen science as a pseudo-science, and overstating of impact). Current practitioner responses and potential future solutions to these limitations will be discussed.

#### Methodological Shortcomings: Self-Reported Data

There are limitations in the methods currently used by researchers in crowdsourced health research studies. Much of the available information is self-reported, and it cannot be verified whether the participant actually has the condition, engaged in the intervention(s), and/or reported accurate outcome data. In addition, disease patients may not be sufficiently reliable to diagnose and report on their own conditions [62]. Study organizers have managed these challenges by conducting in-house calculations to see how results would differ if some of the controls were really cases and vice versa [62], collecting (but not verifying) attending physician information [30], pointing out that it would be time-consuming and without ostensible benefit to participants to falsify data [30], and asking study participants to submit externally-validated data (such as blood test results via PDF forms) [56]. Encouragingly, 1 study found that crowdsourced data was at least as good or better than traditionally collected samples [63]. In the future, statistical analysis and automated data checks could be developed to confirm prescription activity with diagnosing physicians and otherwise validate data, perhaps similar to anomaly detection, credit-scoring, and fraud detection algorithms.

#### Study Design Shortcomings: Protocol, Self-Selection Bias, and Funding

Limitations are also apparent in study design employed in crowdsourced health research studies in the areas of protocol, self-selection bias, and funding. Crowdsourced health research studies do not always follow the rigorous protocols of randomized blinded controlled studies. A variety of alternative designs are the norm in crowdsourced health research; these designs enable costs to remain relatively low and increase feasibility of conducting studies. Designs used include observational studies, crossover studies, and adaptive studies. Some studies are without blinding and/or do not include a placebo arm. Concerns have been raised that differences may be observed when participants are subjects who knowingly collect and report their own data. Therefore, such biases may be particularly pronounced in participant-organized studies; especially where protocols are unclear.

Another protocol limitation has been that, thus far, the only form of study conducted by professional researchers in crowdsourced cohorts has been retrospective, non-interventional user questionnaires. There is, therefore, an opportunity for professional researchers to pursue intervention-based studies in crowdsourced cohorts.

Within crowdsourced studies there is the potential for self-selection bias. Crowdsourced study members are like-minded participants that are perhaps not representative of the target population [62]. This can result in cohort populations that are too homogenous or too heterogeneous, and cohorts that have high intra-individual variance, small sample sizes, and results that are not statistically significant. Self-selection bias could be improved both through alternative study design protocols, for example in the case of genetic studies using a complementary genome-wide association study (GWAS) approach to validate results [62], and through funded collaborations with patient advocacy groups to help recruit larger, more representative cohorts [56].

A criticism has arisen that crowdsourced health research study findings may not be fully accepted if studies are industry-financed; this critique is also made of more traditional studies. The greater transparency available in crowdsourced health research studies could be an asset in the validation of study results as any interested independent party could view study results (both positive and negative) publicly. Cost is a barrier to widespread participation in crowdsourced studies, so standard models for funding that do not influence the integrity of study results need to be established. Overall crowdsourced health research studies could be viewed as complementary to RCTs, offering a fresh perspective and allowing different types of research questions to be asked.

#### Regulation and Oversight

A limitation of crowdsourced health research studies has been that they do not always conform to generally accepted industry practices of research conduct. Recommendations have been made regarding institutional review board approval, consenting processes, data use policies, and communications to potential participants [64,65]. The process for obtaining informed consent should follow standard legal requirements. For example, information provided to participants should be non-technical and easy to understand, and should make clear which data will be published and in what format (eg, de-identified, aggregated). Many crowdsourced health research studies have been attempting to follow these recommendations. However, other studies specifically point out the practical impossibility of traditional compliance mechanisms and have participants consent to their acknowledgement of this. For example, the Harvard Medical School’s Personal Genome Project (PGP) has participants agree to a more open stance on privacy and data use: “the data that you provide as part of the PGP may be used … to identify you as a participant in otherwise confidential genetic research [66].”

Another research standard is that institutional review board (IRB) approval is required and should be obtained before studies begin. This is a potential limitation in crowdsourced studies as it may not allow for the natural adaptation that can occur in self-experimentation studies. Alternative models that provide the same functions of IRBs could evolve as crowdsourced health research studies become more prominent. The roles and responsibilities of IRBs could be disintermediated and fulfilled by separate parties. For example, independent oversight could be preserved through external expert review. Blanket IRB approval could allow broad adaptive investigative research in an area with overall safeguards and restrictions in place. Financial liability could be grounded in group insurance policies for health collaboration communities [18].

#### ‘Novel Findings Do Not Necessarily Flow From Novel Crowdsourced Methodologies’ 

Crowdsourced health research study methods are novel in several ways. First, the research uses Internet-based technologies to contact large numbers of potential study participants. Second, data is often available publicly (or to the study community) such that anyone can search the longitudinally-tracked real-time information in pre-aggregated patient communities; this openness is rarely (if ever) seen in more traditional studies. Although, while crowdsourced research methodologies are novel, it does not necessarily follow that they always beget novel discovery. Some critics argue that the same findings might have occurred with traditional methods. However, crowdsourcing provides a cheaper and faster alternative. In addition, crowdsourced studies can potentially draw on a much larger and more committed base of potential participants. Crowdsourcing studies may be useful in many dimensions including developing novel methods, discovering novel findings, and replicating existing findings in larger groups with more permutations. The key point is that valid scientific findings can occur with crowdsourcing techniques.

#### ‘Citizen Science Is Not Real Science’

In self-experimentation and participant-organized studies citizen scientists may be engaging in science-related activity but their activity (according to formal definitions) might not be regarded as science. The established scientific steps of creating well-formed falsifiable hypotheses, collecting and analyzing data, and publishing peer-reviewed results may not be followed in every project (nor need be followed in every project). The motivations behind individual crowdsourced projects can be different, and projects vary in the quality of crowdsourced science. This variation extends from professional researchers conducting large-scale peer-reviewed studies to individuals engaging in self-experimentation projects for personal knowledge and benefit. While all levels of crowdsourced activity may be useful, some argue that the term *science* should not be applied to projects unless the whole scientific process is followed. One of the benefits of new models like crowdsourcing is enabling a much broader audience to participate in science-related activities. This could help to dispel elitism [67] where science is “a closed society organized into fiefdoms of highly trained specialists” where “only a few minds engage with any problem [68].” Early successes in citizen science have shown “the potential to alter the landscape of science in important ways, harnessing countless able brains to do work that was once the province of a few overwhelmed experts [68].”

#### ‘Overstating the Impact of Crowdsourced Health Research Studies’

The area of crowdsourced health research is charged with overstating the potential growth and impact of participatory health initiatives. Critics argue that not all individuals are interested in health; instead they perceive it as the responsibility of physicians. The interest of individuals could further be undermined by health privacy concerns that impact on crowdsourced health research studies. These barriers currently mean that few individuals engage in health action-taking unless it is easy, automated, and (possibly) accompanied by financial incentives.

#### ‘There Are More Pressing Public Health Challenges Requiring Attention’

Crowdsourced health research studies may seem like an unimportant detail in the overall public health landscape where there is more urgency of focus on the near-term challenges of budget shortfalls, rising health care costs, anticipated physician shortages, aging populations, and the exorbitant cost of bringing new drugs to market (currently estimated at $1.3 billion [69]). However, participatory health initiatives might be able to help in addressing these challenges. They facilitate the realization of a new sensibility of health self-management through crowdsourced projects that could lead to healthier and more engaged populations, and have enduring benefits for both individuals and public health systems.

### Conclusions

#### Growth of Participatory Health

Participatory health initiatives (self-tracking devices, smartphone applications, online personal health records, health social networks, and crowdsourced health research studies) are growing quickly; growth is facilitated by Internet and social networking influences. Individuals are joining researcher-organized studies, and designing and operating studies of their own. It is becoming easier to experiment with the launch of self-monitoring gadgets and smartphone applications, decreasing costs of genomic sequencing, availability of low-cost direct-to-consumer blood tests for testing interventions, increased access to online bioinformatics tools for data interpretation, and the advent of local DIYbio (do-it-yourself biology) labs [67] with facilities for education and experimental projects. Crowdsourced health research studies are emerging as an important new means of investigation in a multi-tier ecosystem that could include self-experimentation, participant-organized crowdsourced studies, and researcher-organized crowdsourced studies that are a complement and precursor to traditional RCTs. Over time, there could be greater convergence between citizen scientists and the established research enterprise. There are exciting opportunities for researcher-organized study activity in health social networks to move beyond survey-based methods towards active intervention-testing.

#### The Bigger Context of Crowdsourcing and Participatory Health: Preventive Medicine

Crowdsourcing has cost and speed benefits; it may allow science to be conducted at scales of magnitude greater than before (thousands recruited in months versus years [43,70] and billions of data points per person [71]), potential novel discovery in the patterns of large data sets [72,73], and the possibility of near real-time testing and application of new medical findings. Larger cohorts and more granular data could enable investigation in a much more detailed range of parameter-stratified sub-cohorts. Crowdsourced participatory health research efforts are helping to expand the conceptual scope of medicine from the traditional focus on disease cure to the personalized preventive medicine of the future.

## Abbreviations

ALS: amyotrophic lateral sclerosis
DIYbio: do-it-yourself biology
GWAS: genome-wide association study
IRB: institutional review board
MS: multiple sclerosis
MTHFR: methylenetetrahydrofolate reductase
PLM: PatientsLikeMe
PGP: personal genome project
RCT: randomized controlled trial
